# A simplified model for predicting the minimum miscibility pressure of CO_2_ flooding in reservoirs of the Ordos Basin

**DOI:** 10.1371/journal.pone.0339648

**Published:** 2026-01-08

**Authors:** Wei Wang, Qinglong Zhu, Jian Liu, Zhenjie Yao, Shiyu Wang, Peihao Xu

**Affiliations:** 1 Shaanxi Key Laboratory of Carbon Neutral Technology, Carbon Neutrality College, Northwest University, Xi’an, Shaanxi, China; 2 Research Institute of Exploration and Development, PetroChina Changqing Oilfield Company, Xi’an, Shaanxi, China; 3 Research Institute of Shaanxi Yanchang Petroleum (Group) Co., Ltd., Xi’an, Shaanxi, China; China University of Petroleum Beijing, CHINA

## Abstract

Minimum miscibility pressure (MMP) is a critical parameter in reservoir screening and development design for CO_2_ flooding projects. With the expanding implementation of CO_2_ flooding in the Ordos Basin, practical challenges have emerged, including difficulties in acquiring crude oil samples and the high cost of testing during the screening phase. Through experimental measurements and theoretical analysis, significant compositional similarities among different crude oils were revealed. Based on this finding, a simplified MMP prediction model was developed, requiring only reservoir temperature as input. Among the evaluated models, the quadratic polynomial model achieved the highest fitting accuracy (R^2^ = 0.841) and the smallest root mean square error (RMSE). Validation using oil samples from a candidate block for CO_2_ flooding yielded absolute and relative errors of 0.93 MPa and 4.93%, respectively, indicating that the model satisfies the accuracy requirements for MMP prediction and miscibility assessment in reservoir screening. The proposed method provides robust technical support for the large-scale deployment of CO_2_-enhanced oil recovery and storage in the Ordos Basin.

## 1. Introduction

The Ordos Basin, a principal energy and chemical industry base in China, contains abundant reserves of oil, gas, and coal resources [1]. Against the backdrop of global climate change and the national “carbon peaking and carbon neutrality” strategy, there is an urgent need to explore low-carbon and efficient approaches for the development and utilization of fossil energy. CO_2_ emissions from the energy and chemical industry can be effectively stored in the basin’s low-permeability reservoirs. This not only alleviates regional CO_2_ emission reduction pressures [2,3] but also substantially enhances the recovery of low-permeability oil and gas reservoirs [4,5], thereby achieving the dual objectives of emission reduction and increased production. The Triassic Yanchang Formation in the central–western Ordos Basin is the most promising target interval for CO_2_-EOR and storage. According to current assessments, this formation has a storage potential exceeding 1.91 billion tons [6], indicating substantial prospects for large-scale application. In recent years, Yanchang Petroleum and Changqing Oilfield have successfully implemented pilot and demonstration projects in blocks such as Jingbian Qiaojiawa, Ansai Huaziping, and Jiyuan Huang-3 [2,7,8]. With planned project capacities already surpassing tens of millions of tons, CO_2_-EOR and storage technologies are entering a stage of accelerated deployment across the basin [8].

CO_2_ flooding can be categorized into miscible and immiscible processes, a distinction determined by the relationship between reservoir pressure and the minimum miscibility pressure (MMP). When reservoir pressure exceeds the MMP, crude oil and CO_2_ form a single phase, achieving maximum displacement efficiency. When the pressure lies within 0.8–1.0 times the MMP, although full miscibility is not achieved, this state is defined as near-miscible flooding [9]. As the miscibility increases, the CO_2_ front moves more stably and sweeps a larger area [10], leading to increased CO_2_ storage range and volume. Consequently, accurate determination of MMP between reservoir oil and CO_2_ is essential not only for identifying the miscible state, but also for reservoir screening, potential evaluation, development planning, and injection-production optimization in CO_2_-EOR and storage projects.

Currently, methods for determining MMP can be broadly classified into three categories: (i) experimental techniques such as slim-tube test, rising bubble apparatus, and interfacial tension measurements [11]; (ii) theoretical calculations based on fluid phase behavior models or molecular dynamics simulations [12,13]; (iii) empirical correlations derived from regression of experimental data [14–16]. However, both experimental testing and theoretical calculation approaches typically require crude oil samples from target reservoirs, as experimental methods necessitate determination of physical property parameters such as MMP, crude oil composition, and phase behavior. During the CO_2_-EOR reservoir screening phase, large-scale application of these methods faces challenges including high sampling demand, long testing cycles, and substantial costs. This limitation is especially acute in undeveloped reservoirs, where the absence of oil samples often renders conventional methods infeasible. Although empirical correlations, which establish mathematical relationships between MMP and parameters such as temperature and oil composition, have significantly improved predictive efficiency, their general applicability remains constrained by the need for detailed compositional data.

Analyzing crude oil compositional data from representative reservoirs in the Ordos Basin, this study reveals high similarity in crude oil composition across different stratigraphic intervals. Based on this finding, a simplified MMP prediction model using reservoir temperature as the sole input parameter was developed. This model effectively addresses the challenges of conventional methods for large-scale CO_2_-EOR reservoir screening, such as the difficulty of obtaining oil samples and the high costs of laboratory testing and analysis. Thus, the proposed model provides a reliable tool for rapid screening of target reservoirs and accurate determination of miscibility conditions for CO_2_ flooding projects within the basin.

## 2. Factors controlling the MMP of CO_2_ flooding

Studies have shown that the factors influencing the MMP include the reservoir temperature, crude oil composition, CO_2_ purity, and pore radius [17].

### 2.1. Reservoir temperature

It is well-established [18,19] that under typical reservoir temperature and pressure conditions, higher temperatures decrease CO_2_ solubility in crude oil, thereby increasing the minimum miscibility pressure (MMP). For instance, Yellig et al. [20] reported that the MMP for crude oils from west Texas increased at a rate of 0.057 MPa/°C with rising temperature, indicating the significant influence of reservoir temperature on MMP.

**Fig 1:**
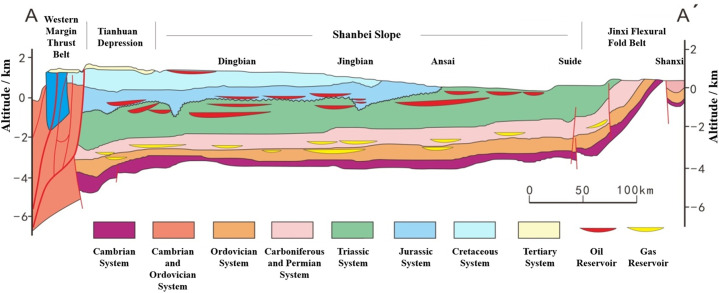
Schematic diagram of the Northern Shaanxi slope cross-section in the Ordos Basin. The map shows the location of the studied reservoirs.

The Ordos Basin has a simple geological structure, and the formation temperature increases linearly with increasing depth, exhibiting characteristics typical of a conductive geothermal field [21]. Although reservoir burial depth and temperature vary across blocks-with most reservoir temperatures ranging between 40°C and 100°C-the simple geological structure (Fig 1) ensures a largely consistent geothermal gradient of approximately 2.75 to 2.86°C/100m [22]. Consequently, reservoir temperature, which is readily obtainable from well logging data or geothermal gradient calculations, stands out as the most accessible and practical parameter for MMP estimation.

### 2.2. Reservoir oil composition

Crude oil composition is another critical determinant of MMP [23,24]. Alston et al. [25] demonstrated that C_2_-C_4_ components, gaseous at ambient temperature but with supercritical temperatures higher than that of CO_2_, can effectively reduce MMP between crude oil and CO_2_. Orr [26] and Zhang et al. [27] revealed that the MMP between pure C_5_+  hydrocarbon components and CO_2_ increases with both carbon number and molecular structure complexity, though carbon number exerts greater influence. Consequently, existing empirical MMP correlations typically categorize crude oil composition into CH_4_, intermediate components, and heavy components. Depending on the specific correlation, heavy components are commonly defined as C_5_+, C_7_+, C_10_+, or C_20_+ [19,28]. Generally, higher concentrations of CH_4_ and heavy components in crude oil hinder miscibility, while a greater content of intermediate components promotes it. However, obtaining the accurate in-situ compositional data required by these correlations presents a major bottleneck. The process necessitates specialized downhole fluid sampling and extensive laboratory analysis, which are hindered by operational complexities and prohibitive costs.

**Fig 2:**
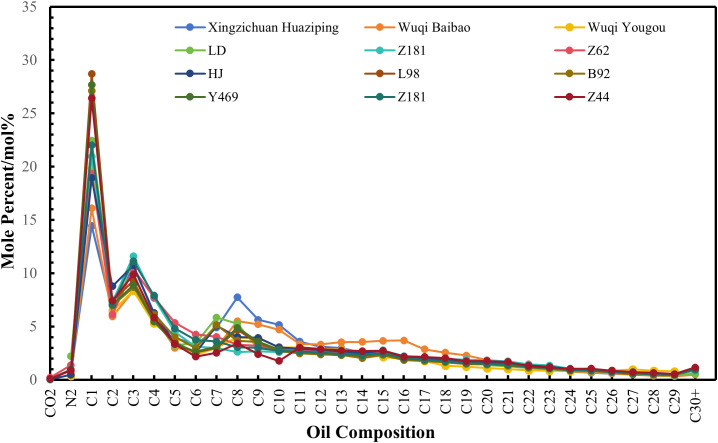
Composition of live oil from different reservoirs in the Yanchang formation, Ordos Basin. The chromatograms of 12 crude oil samples show consistent peaks at C_1_, C_3_, and C_7-8_, indicating high compositional similarity.

To achieve efficient oil displacement and high-density storage using supercritical CO_2_, the CO_2_ must maintain a supercritical state under reservoir conditions (temperatures ≥31.1°C, pressures ≥7.38 MPa). For the Ordos Basin, this translates to suitable CO_2_ flooding reservoirs being primarily distributed in Triassic formations at depths exceeding 900 meters in the central-western regions. Major target layers include the Chang 4+5, Chang 6, Chang 8, Chang 9, and Chang 10 sub-layers. The crude oil in these layers predominantly originated from the Chang 7 and Chang 9 source rocks [29–31]. While the Chang 4+5 and Chang 6 layers are widely believed to derive from the Chang 7 source rock [32,33], the origin of the Chang 8, Chang 9, and Chang 10 layers remains debated, with proposed sources including the Chang 7 [32–34], Chang 9 [35], or a mixture of both [30,31]. This debate indirectly suggests minor differences in crude oil properties across these layers. Liu [29] and Guo et al. [36] explicitly reported that the physical properties and group composition of crude oils from various Yanchang Formation layers are essentially identical, all classified as light oils with low density, viscosity, and pour point. Corroborating these findings, our analysis of 12 crude oil samples from the basin (Fig 2) reveals that the composition curves of oils from different reservoirs and layers exhibit remarkable similarity, characterized by consistent peaks at C_1_, C_3_, and C_7-8_. Combining with existing understanding of oil sources and comprehensive component analysis, these results demonstrate strong compositional similarity among crude oils from various reservoirs and layers across the basin. Therefore, the MMP prediction model developed based on slim-tube test data from different reservoirs within the basin effectively mitigates the influence of crude oil composition variations on MMP values.

### 2.3. CO_2_ purity

**Fig 3:**
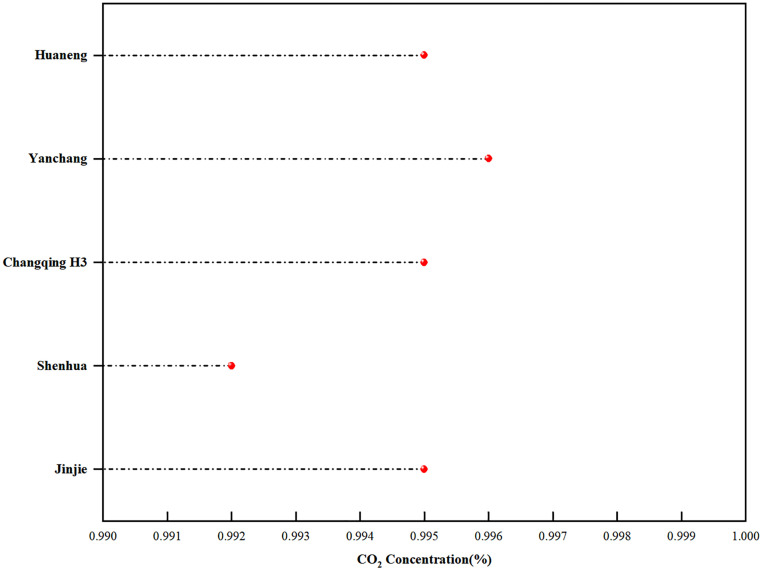
Comparison of CO_2_ capture concentrations in the Ordos Basin. The data presented in the figure indicate that the injected CO_2_ for enhanced oil recovery across the basin has a purity exceeding 99.5%, with the exception of the Shenhua saline aquifer project, which has a purity of 99.2%.

The CO_2_ injected into reservoirs primarily originates from captured anthropogenic CO_2_ or natural CO_2_ gas reservoirs. Its purity is often affected by two types of components: (i) inherent impurities from gas sources, such as N_2_, H_2_S, CH_4_, CO, and light hydrocarbons; (ii) performance-enhancing additives, including light hydrocarbons, low-carbon alcohols, petroleum ether, and surfactants, which are introduced to mitigate gas channeling or improve miscibility [37]. These impurities or additives significantly affect the MMP between CO_2_ and crude oil. Specifically, non-condensable gases like N_2_ and CH_4_ tend to increase MMP, whereas H_2_S, light hydrocarbons, petroleum ether, low-carbon alcohols, and surfactants contribute to MMP reduction. However, the MMP data employed in this study were obtained from slim-tube tests using CO_2_ with purity exceeding 99.9% [8]. As shown in Fig 3, the CO_2_ injected for enhanced oil recovery in basin reservoirs demonstrates high purity, typically exceeding 99.5% [38–40], with the saline aquifer storage Shenhua project being an exception at 99.2% [41]. Thus, the effect of CO_2_ purity on MMP can be neglected in the MMP prediction model.

### 2.4. Pore radius

Early studies seldom accounted for the effect of reservoir rock pore-throat size on MMP. However, recent advances in microscale confinement theory have revealed distinct fluid phase behaviors in microscopic systems compared to conventional bulk systems. This discrepancy stems from the interplay between intermolecular forces and fluid-rock wall molecular interactions, which can potentially reduce CO_2_-crude oil MMP in nanoscale pore throats [42]. Zhang et al. [17] and Wu et al. [42] suggested that MMP can be reduced in pore throats below 100 nm, whereas Fang et al. [13] and Teklu et al. [43] argued that reduction only occurs below 15–20 nm, indicating ongoing scholarly debate. Although the Triassic reservoirs in the Ordos Basin typically exhibit ultra-low permeability, with pore-throat size predominantly ranging from 60 to 900 nm [44], the empirical correlation developed in this study is based on MMP data from Darcy-scale slim-tube tests [8]. Since these experiments did not consider microscopic pore-throat effects, such influences of pore-throat size are excluded from the present work.

## 3. Simplified MMP prediction model

### 3.1. Model development

The preceding analysis establishes reservoir temperature as the dominant controlling factor for CO_2_ MMP in the Ordos Basin, particularly for undeveloped reservoirs where compositional data are scarce. This finding justifies the development of a simplified prediction model that relies solely on temperature. By reviewing existing MMP prediction models [14,17,19,45] and holding constant all parameters except temperature, we derived six functional relationships between reservoir temperature and MMP: linear, power, logarithmic, quadratic polynomial, Zhang model, and Lee model. Additionally, during the fitting process, we found that a logistic model yielded a relatively high coefficient of determination for slim-tube experimental data relating reservoir temperature to MMP in the Ordos Basin. In summary, the functional expressions for the seven models are detailed in Table 1.

**Table 1:** Comparison of different MMP prediction models and their fitting results.

Prediction Model		Fitting Parameters and Results					
Model Type	Model Formula	a	b	c	d	R^2^	RMSE
**Linear**	MMP_pre_ = a*T + b	0.1442	8.4210	/	/	0.7187	1.3305
**Power**	MMP_pre_ = a*T^b	2.0268	0.5221	/	/	0.7476	1.2601
**Logarithmic**	MMP_pre_ = a*ln(T)+b	9.6101	−22.0758	/	/	0.7753	1.1891
**Zhang**	MMP_pre_ = a*(ln(b*T + c))^d	16.6209	0.1098	−3.0941	0.3342	0.8230	1.0554
**Lee**	MMP_pre_ = a + b*exp(c*T^d)	20.6759	−36.3273	−0.0052	1.5192	0.8344	1.0208
**Logistic**	MMP_pre_ = a/(1 + b*exp(c*T))	20.6353	17.2952	−0.0794	/	0.8347	1.0197
**Quadratic**	MMP_pre_ = a + b*T + c*T^2	−8.7524	0.6855	−0.0040	/	0.8408	1.0007

Notes: In the table, MMP_pre_ refers to predicted minimum miscibility pressure (MPa); T denotes reservoir temperature (°C).

### 3.2. Model fitting

**Fig 4:**
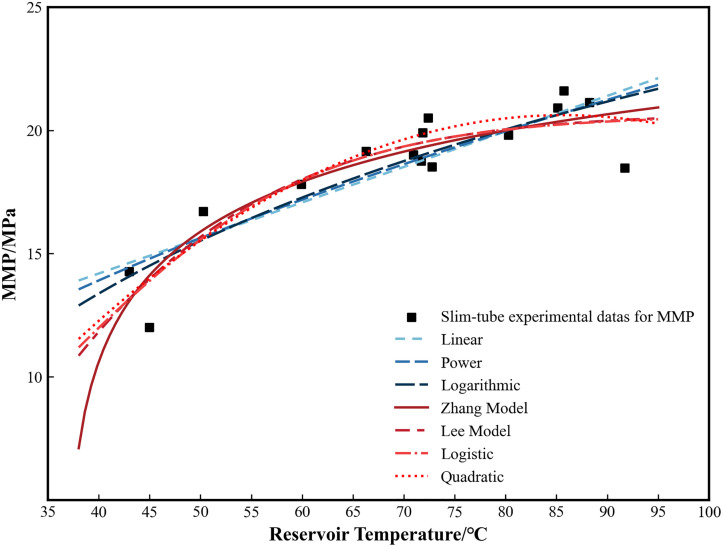
Comparison of fitting curves of different MMP models. The quadratic polynomial model shows the highest R^2^ and lowest RMSE among all evaluated models.

A total of 15 sets of MMP slim-tube test data for reservoir oil and CO_2_ from various Ordos Basin reservoirs were compiled in this study (Table 2). These data were sourced from existing literature (13 sets) [8,46–49] and technical reports (2 sets). As all data were from pre-existing sources, no permits for field site access were required for this work. Based on this dataset, seven MMP prediction models were fitted as functions of temperature. The corresponding coefficients and performance metrics are presented in Table 1 and Fig 4. The coefficient of determination (R^2^) and root mean square error (RMSE) in Table 1 represent the goodness-of-fit and deviation, respectively. Higher R^2^ values coupled with lower RMSE values indicate better agreement between model predictions and experimental data.

**Table 2:** Experimental and predicted values of MMP for crude oil-CO_2_ systems in some reservoirs.

Block	Reservoir Stratum	Temperature /°C	Original Formation Pressure /MPa	MMP_slim-tube_ /MPa	MMP_pre_ /MPa	Miscible State
Xingzichuan Huaziping [8]	Chang6	43.00	8.90	14.27	13.24	Immiscible
Wuqi Baibao [46]	Chang9	72.80	17.50	18.52	19.70	Near-miscible
Wuqi Yougou [47]	Chang4+5	59.94	13.30	17.80	17.80	Immiscible
Ansai Wangyao [48]	Chang6	45.00	9.13	12.00	13.90	Immiscible
Ansai Xinghebei ^a^	Chang6	50.30	10.20	16.70	15.49	Immiscible
Xifeng Baimaqu [49]	Chang8	66.30	18.10	19.14	18.90	Near-miscible
Jiyuan Luo1bei ^a^	Chang8	80.30	19.00	19.80	20.20	Near-miscible
H138 [46]	Chang8	91.73	19.74	18.47	20.07	Miscible
Z62 [46]	Chang8	71.73	18.98	18.75	19.60	Miscible
HJ [46]	Chang8	88.25	21.54	21.12	20.22	Miscible
L98 [46]	Chang8	85.12	19.99	20.90	20.27	Near-miscible
B92 [46]	Chang8	71.85	17.92	19.90	19.61	Near-miscible
Y469 [46]	Chang8	72.42	17.80	20.49	19.67	Near-miscible
Z181 [46]	Chang8	70.94	22.36	19.00	19.51	Miscible
Z44 [46]	Chang8	85.74	20.01	21.60	20.27	Near-miscible

Notes: (1) The data marked with ‘a’ are from the “Report on the Work of CO_2_ Flooding in Changqing Oilfield,” September 2014. (2) The miscibility classification criteria are as follows: a miscibility index > 1.0 is defined as miscible, an index between 0.8 and 1.0 is considered near-miscible, and an index < 0.8 is classified as non-miscible.

The results show that the linear, power, and logarithmic functions exhibit a poor fit to the experimental data, with R^2^ values below 0.80. In contrast, the Zhang, Lee, Logistic, and quadratic polynomial models demonstrate a superior fit, all achieving R^2^ > 0.82. Based on the P-T phase diagram of the CO_2_-crude oil system [45] and established phase-behavior theory [50], the MMP increases with temperature at lower ranges, although the rate of increase progressively diminishes, indicating a reduced tendency for miscibility at higher temperatures. Once the temperature exceeds a critical high-temperature threshold, the CO_2_-crude oil system transitions to a gas-gas state and attains miscibility [51], leading to a decrease in MMP with further temperature rise. Under such conditions, elevated temperatures instead promote miscibility. The linear, power, and logarithmic models exhibit MMP increasing too rapid with temperature, contradicting the phase behavior characteristics of the CO_2_-crude oil system. The remaining four models are consistent with theoretical understanding: although MMP increases with temperature, the rate of increase decelerates. Notably, the quadratic polynomial model achieves the highest R^2^ (0.841) and the lowest RMSE, indicating superior fitting accuracy. Its curve morphology matches the globally recognized trend: MMP initially increases with temperature, but after reaching an extremely high crude oil vaporization temperature, it decreases with further temperature rise [51].

**Fig 5:**
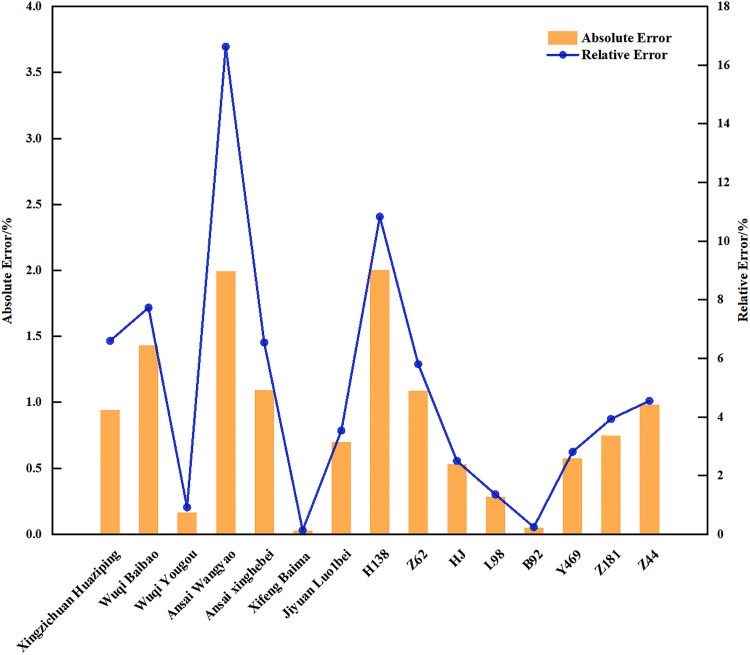
Analysis of fitting error of secondary polynomial MMP prediction model. The bar chart represents the absolute error, and the dotted line with markers represents the relative error.

To further assess the fitting error of the quadratic polynomial MMP prediction model, predicted and experimental MMP values from different blocks were compared in Table 2 and Fig 5. The results demonstrate high accuracy: although the maximum absolute error reaches 2.00 MPa in the Ansai Wangyao block, the average absolute and relative errors across all datasets are 0.84 MPa and 4.94%, respectively, which are well within engineering tolerances. Therefore, the quadratic polynomial model is the preferred preliminary choice for rapid screening of CO_2_ flooding reservoirs in the basin, based on data fitting results. The formula is: MMP_pre_ = −0.004 T^2^ + 0.6855 T – 8.7524.

### 3.3. Model Verification and Application

To validate the accuracy of the newly quadratic polynomial MMP prediction model for CO_2_ flooding MMP estimation in reservoirs of the Ordos Basin, crude oil samples were collected from Block X—a developed block adjacent to several new CO_2_ flooding candidate areas in Changqing Oilfield. This block produces from the Chang 8 reservoir layer, at 71.4°C reservoir temperature and 18.50 MPa original formation pressure. For this sample, slim-tube testing yielded an MMP of 18.87 MPa, while the model predicted 19.80 MPa, corresponding to an absolute error of 0.93 MPa and a relative error of 4.93%. These results demonstrate high prediction accuracy.

**Fig 6:**
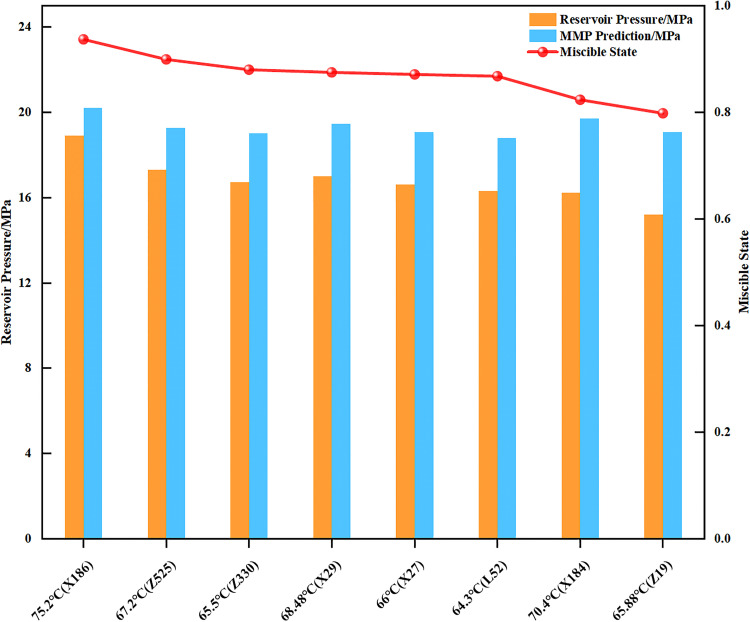
The prediction of MMP values for new areas to be screened for CO_2_-EOR in a certain region of the Changqing Oilfield. The blue and orange bars represent the predicted minimum miscibility pressure (MMP) and the original reservoir pressure for each candidate block, respectively. The dotted line indicates the corresponding miscibility degree (Pres/MMP).

The newly quadratic polynomial model was applied to estimate the CO_2_ flooding MMP for several candidate blocks adjacent to Block X, with the results detailed in Fig 6. Analysis shows that for all blocks, predicted MMP values exceed original reservoir pressures, yielding miscibility (P_res_/MMP) of 0.80−0.94. This indicates that CO_2_ flooding under original reservoir pressure conditions would achieve a near-miscible stage in these areas. Implementing CO_2_ flooding strategies tailored to different miscibility conditions allows for an effective balance between efficiency and cost, thereby providing a reliable basis for rapid screening and quantitative decision-making for CCUS projects in the Ordos Basin. However, the preliminary prioritization of candidate reservoirs for CO_2_ flooding, derived from the available data, should be further refined by incorporating evaluations of reservoir properties and caprock integrity. This refinement ensures a more comprehensive reservoir selection, directly addressing the high-cost and sample-scarcity challenges in large-scale screening mentioned.

## 4. Conclusion

(1) Analysis of MMP-controlling factors identified striking compositional similarity among crude oils from Triassic reservoirs in the Ordos Basin. Given the temperature’s significant impact and data accessibility, this study proposes a simplified MMP prediction model with reservoir temperature as its sole input parameter, tailored for the basin.
(2) Based on regression analysis of 15 sets of CO_2_-crude oil slim-tube experimental data, the quadratic polynomial MMP prediction model (MMP_pre_ = −0.004T^2^ + 0.6855T - 8.7524) yielded the highest accuracy (R^2^ = 0.841, lowest RMSE) among seven temperature-dependent models. Furthermore, its predictive curve aligns with the phase behavior evolution laws of the CO_2_-crude oil system.
(3) Validation with CO_2_-crude oil MMP data from Block X in the Ordos Basin showed that the quadratic polynomial prediction model yielded absolute and relative errors of 0.93 MPa and 4.93%, respectively. This accuracy meets the requirements for rapid MMP determination and miscible state evaluation in new CO_2_ flooding target reservoirs, addresses the challenges of sample scarcity and high testing costs during the screening phase, and thus supports reservoir selection for CCUS projects in the Ordos Basin.
